# Vitamin D deficiency prevention policies in Iran: a retrospective policy analysis

**DOI:** 10.3389/fnut.2023.1249402

**Published:** 2023-08-23

**Authors:** Baharak Aghapour, Sorayya Kheirouri, Mohammad Alizadeh, Rahim Khodayari-Zarnaq

**Affiliations:** ^1^Department of Community Nutrition, School of Nutrition and Food Sciences, Tabriz University of Medical Sciences, Tabriz, Iran; ^2^Department of Nutrition, Faculty of Nutrition and Food Sciences, Tabriz University of Medical Sciences, Tabriz, Iran; ^3^Department of Health Policy and Management, School of Management and Medical Informatics, Tabriz University of Medical Sciences, Tabriz, Iran

**Keywords:** vitamin D deficiency, agenda setting, Kingdon's multiple streams, prevention, vitamin D

## Abstract

**Aim:**

Iran has a higher prevalence of vitamin D deficiency (VDD) than the global level. This study aimed to assess VDD prevention policies in Iran through a policy analysis of agenda setting using the multiple streams framework (MSF).

**Methods:**

Using Kingdon's MSF model, this qualitative analytical study performed a policy analysis on vitamin D-related policies in Iran. The policy documents were reviewed, and in-depth interviews were conducted with stakeholders (*n* = 27) using the framework analysis method. To categorize data and extract the related themes, MAXQDA version 10 was used.

**Results:**

According to Kingdon's MSF theory, the problem stream included the high prevalence of VDD among Iranian infants (23.3%), adolescents (76%), and adults (59.1%). The policy stream was identified to focus on preventing programs for non-communicable diseases in the health sector. The political stream indicated that national and international support could provide a political climate for this issue.

**Conclusion:**

According to our results, a window of opportunity for policymaking on VDD prevention has opened. However, there are some challenges related to the implementation of these policies. These include the dominance of a treatment-based view rather than a prevention-based approach in the health sector, economic problems, and restricted access to health services due to the outbreak of coronavirus disease 2019 (COVID-19). To strengthen and implement VDD prevention policies, the stakeholders need support from high-level policymakers.

## 1. Background

Vitamin D deficiency (VDD) is considered a global public health issue associated with many chronic diseases, including cancers, diabetes, multiple sclerosis, immune system dysfunction, and cardiovascular diseases (1–3). However, there is a dearth of knowledge on VDD in many countries, particularly in low- and middle-income ones. Moreover, there are many ways to measure 25-hydroxy vitamin D (25(OH)D), which makes it challenging to integrate the findings (4–6). The results of the second National Integrated Micronutrient Survey (NIMS) in Iran showed that the prevalence of VDD was 23.3, 76, 59.1, and 85.3% in infants aged 15–23 months, adolescents, adults, and pregnant women, respectively (7). Other studies have also established that the prevalence of VDD is high among Iranian pregnant women and children (8). The economic burden of the diseases attributed to hypovitaminosis D could be markedly reduced in the community by improving vitamin D levels to the optimal level (9, 10).

Uday et al. reported that vitamin D supplementation programs are implemented for infants in 96% of European countries, though the commencement time, duration, and implementation method vary among different countries (11). The results of an Iranian study indicated that the national vitamin D supplementation program is a cost-effective way to manage cardiovascular diseases among adults (12). Given that it is necessary during infancy and childhood (13), vitamin D supplementation is being implemented as a preventive strategy in all children under 2 years old in Iran (14).

Since 2014, a 9-month supplementation program with a mega dose (50,000 IU/month) of vitamin D has been carried out among all Iranian high school students monthly, prioritizing regions with the highest deficiency. Adolescents, adults, and older adults who visit health clinics are also covered by similar national laws, which mandate the provision of a vitamin D supplement of 50,000 IU per month. Similarly, pregnant women are recommended an intake of 1,000 IU of daily vitamin D.^1^ Along with the supplementation program, nutrition education is also organized for different groups (15).

To make the policies effective, it is necessary to adapt policies from the agenda-setting stage as the first step of the policymaking process. Despite the significant incidence of vitamin D insufficiency in many nations throughout the globe, limited comprehensive studies have been carried out on VDD preventive policies in low- and middle-income countries. To address this gap, we used the Kingdon's multiple streams framework (MSF) model in this study to identify the existing streams and explore how policymakers placed hypovitaminosis D prevention policies as a political priority on the agenda in Iran.

### 1.1. Conceptual framework

In this study, we chose the Kingdon's MSF model, which is widely used by researchers and policymakers in agenda-setting. MSF is the first step in the policymaking process and can be divided into three separate parts, including problem stream, policy stream, and political stream (16). This model focuses on the role of key policy stakeholders inside and outside the government and seizes streams to create opportunities called “policy windows.” This framework determines “what were the problem streams?” “what were the policy streams?” “what was the politics stream at that time?” and finally, “what made the window of opportunity open?”

**Figure 1:**
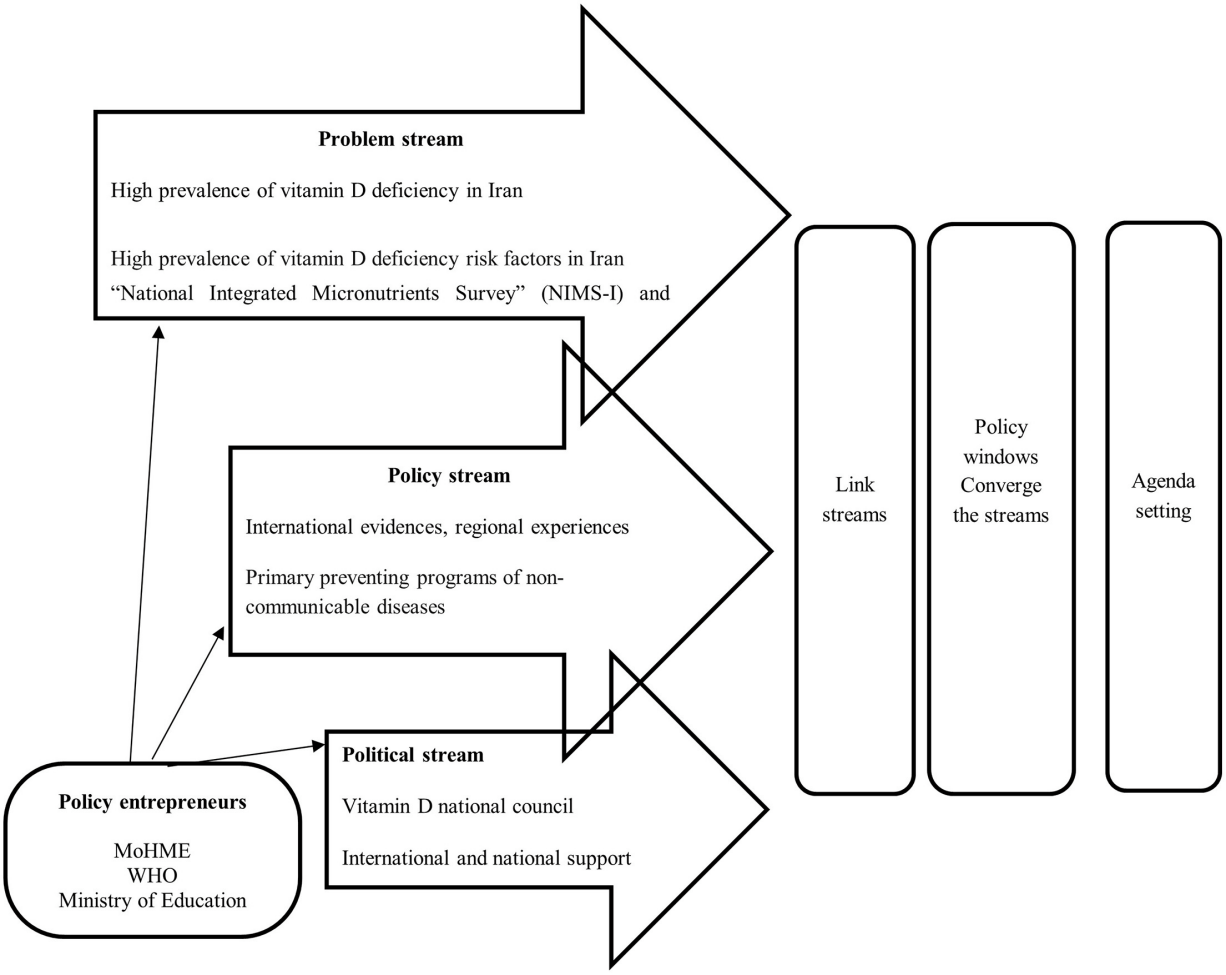
Vitamin D agenda-setting in Iran based on the Kingdon's multiple streams framework.

Based on Kingdon's model, as the three streams (i.e., problem stream, policy stream, and political stream) converge, the issue is considered by policymakers on the agenda more seriously. The problem stream, which includes policy reports, data indicators, and pressure from advocacy organizations, describes how the issue is considered by policymakers. The policy stream clarifies various ideas competing for acceptance and proposes a set of solutions to solve the problem. The chance of accepting the ideas can induce technical feasibility and acceptance of values. Finally, the political stream refers to the national and international climates, as well as political factors affecting the rise of an issue on the agenda. Based on this framework, when the three streams converge at critical time points, a “window of opportunity” will open (Figure 1) (17, 18).

## 2. Material and methods

This qualitative study used an MSF-based approach to explore the main factors affecting the VDD problem stream, solutions to prevent hypovitaminosis D, and the political situations influencing the emergence of VDD prevention policies in Iran.

### 2.1. Documents review

We explored all the available documents related to vitamin D policies, including policy documents, laws, scientific and national studies, newspaper articles, regulations, and government reports. An initial search was conducted in the available governmental reports, the web pages of the Ministry of Health and Medical Education (MoHME), Iranian government agencies, Iranian medical universities, and related research sites.

### 2.2. Key informant interviews

Using purposeful and theoretical sampling techniques, semi-structured interviews were carried out by Ph.D. students from Food and Nutrition Policy at the interviewees' workplace. Interviews continued up to data saturation. The interviewer explained the research objectives and refrained from any bias or prejudice during the interview. The key informants and actors were interviewed based on a conceptual framework guideline. The baseline topic guide of the study was pre-tested, and necessary changes were made. Totally, 27 interviews were undertaken with 13 stakeholders from different levels of the MoHME, 4 of whom were academics and researchers, 2 were from the National Nutrition Food Technology Research Institute, and 1 was from the Food and Drug Organization. The other 7 participants were from different levels of the MoHME. Twenty-four interviews were conducted face-to-face, and three interviews were conducted over telephone due to COVID-19 restrictions. After obtaining informed consent from the participants, we recorded (audio only) all interviews, which were transcribed verbatim. The interviews (average time: 34 min) were conducted at the participants' offices between 1 December 2021 and 31 May 2022.

The following questions were asked:

1. **Problem stream:** What are the problems related to vitamin D policies in Iran? How did VDD enter the agenda?
2. **Policy stream:** What is the setting addressed by different organizations to solve VDD issues in Iran?
3. **Political stream:** How do political determinants affect vitamin D prevention policies in Iran?

### 2.3. Data analysis

Using framework analysis, two researchers analyzed the collected materials. Then, the researchers read the transcribed interviews and reviewed the documents several times. To extract the related themes, all content was open-coded and categorized by two authors independently. Any disagreements were resolved through discussion by two researchers (BA and RK). The themes retrieved based on MSF problem identification, policy solutions, and political opportunities were addressed. Data was analyzed by MAXQDA software version 10.

### 2.4. Ethical issues

The ethics committee of Tabriz University of Medical Sciences, Tabriz, Iran approved the study protocol (Code: IR.TBZMED.REC.1400.653). All participants signed a consent form before the interview and had the right to leave the study at any stage. Further, the interviewees' quotes were anonymized, and their job position anonymized.

## 3. Results

The results were structured based on Kingdon's MSF model. Table 1 shows the findings of reviewing 11 policy documents and conducting interviews with 27 key informants with an average work experience of 21 years.

**Table 1:** Professional background of participants.

**Ministry**	**Stakeholder's department**	**Participant's No**.	**Education**	**Jurisdiction level**	**Related role**
MoHME^*^	The office of the community nutrition improvement	P1	Masters in nutrition	National	Expert in charge of prevention and control of micronutrients
	The office of the community nutrition improvement	P2	Ph.D. in nutrition	National	Expert of the community nutrition improvement office
	Department of health	P3	Masters in nutrition	National	Expert from Community Nutrition Improvement Department of University of Iran
	Department of health	P4	Masters in nutrition	National	Expert from Community Nutrition Improvement Department of University of Tehran
	Population and family health department	P5	Medical Degree with specialization in Pediatrics	National	Official from the Department of Population and Family Health
	Health and adolescent department	P6	Ph.D. in nutrition	National	Expert from Health and Adolescent department
	Non-communicable diseases department	P7	Medical Degree	National	Expert from Non-communicable diseases Department
	Institute of nutritional research and food industries of iran	P8	Ph.D. in nutrition	National	Expert from Nutritional Research Laboratory
	Institute of nutritional research and food industries of iran	P9	Professor of the university- Ph.D. in nutrition	National	Expert from of Nutrition Research center
	Endocrine department	P10	PhD in endocrinology and metabolism	National	Expert from Endocrine department
	University of medical sciences	P11	Ph.D. in nutrition	National	Researcher in the field of vitamin D
	University of medical sciences	P12	Ph.D. in nutrition	National	Researcher in the field of vitamin D
	University of medical sciences	P13	Ph.D. in nutrition	National	Researcher in the field of vitamin D
	University of medical sciences	P14	Ph.D. in nutrition	National	Researcher in the field of vitamin D
	The office of the community nutrition improvement of province	P15	Masters in nutrition	Regional	Expert in charge of the community nutrition improvement office of the provincial health center
	The office of the community nutrition improvement of county	P16	Bachelor of nutrition	Regional	Expert of the office of community nutrition improvement of the city health center
	Provincial health center	P17	Bachelor of nutrition	Regional	Health center nutrition expert
	Provincial health center	P18	Bachelor of Nursing	Regional	Health care providers of health center
	Provincial food and drug office	P19	Bachelor of Nursing	Regional	Provincial pharmaceutical affairs expert
Ministry of education	Health and wellness department	P20	Ph.D. in psychology	National	Advisor to head of health and wellness office - ministry of education
	General department of education of Tehran province	P21	Ph.D. in physical education	National	Expert from Ministry of Education
	Ministry of education	P22	Ph.D. in physical education	National	Executive manager in the ministry of education
	General department of education of Tehran province	P23	Bachelor of health and wellness	Regional	Health care and wellness expert
	School	P24	Bachelor of midwifery	Regional	School health coach
	School	P25	Bachelor of psychology	Regional	School health coach
	Health center	P26	Medical Degree	Regional	General practitioner of the health center
MoHME^*^:	Food and drug organization	P27	Master in nutrition	National	Fortification expert

MoHME^*^: Ministry of Health and Medical Education.

### 3.1. Problem stream

#### 3.1.1. High prevalence of VDD in Iran

The interviewees believed that the high prevalence of hypovitaminosis D in Iran was due to people's insufficient exposure to sunlight and limited intake of foods rich in vitamin D. Proponents advocating the prevention of micronutrient deficiency in Iran argue that VDD can impose economic costs due to its association with non-communicable diseases (NCDs). In this regard, one interviewee from the Nutrition Research Institute at the policymaking level stated:

“It is no exaggeration to say that the largest volume of recent studies in the field of micronutrients is related to vitamin D. In terms of the fact that the criterion they had for VDD two decades ago was quite different from now, it is plausible that one reason for the increase in the prevalence of VDD is related to the change in the criterion.” (Participant 8)

Another key informant said:

“Since vitamin D does not have significant food sources in the food basket of most Iranians, and the people's exposure to sunlight is low, the prevalence of VDD is higher. Of course, the same wavelength that synthesizes vitamin D in the skin leads to many skin diseases, including skin cancer.” (Participant 10)

The results of the national surveys on micronutrient status in Iran in 2001 and 2012 were in line with the statements made by our participants. The first national survey was conducted in 2001 to explore the status of micronutrients based on the first National Integrated Micronutrients Survey (NIMS-I) in Iran.^2^ The second National Integrated Micronutrient Survey (NIMS-II) was conducted in 2012 and aimed to assess the nutritional status of four micronutrients, including iron, zinc, vitamin A, and vitamin D (7). According to the reported results, the prevalence of VDD was 23.3% in infants aged 15–23 months and 76% in adolescents. Further, the prevalence of VDD in girls was higher than in boys; approximately 93.9% of girls had vitamin D deficiency. Finally, the prevalence of VDD deficiency was more than 50% in other groups including elderly, adults, and 2–8 year old children, and the highest prevalence of VDD (85.3%) was related to pregnant mothers.^2^

The results of the interviews showed that despite the implementation of the national vitamin D supplementation program in Iranian high schools since 2014, VDD was still common among adolescents and adults. The interviewers explained that one possible reason for VDD was that the vitamin D supplementation program was implemented alongside the iron supplementation program among adolescents in schools. In this program, the executive administrators of the iron supplementation program also administered vitamin D supplementation, and they were not paid extra for their new duties, so they were not motivated enough to properly implement the vitamin D supplement plan. In addition, the outbreak of COVID-19 caused restrictions in the proper implementation of the vitamin D program.

“We are facing a lack of funds and health care workers. Sometimes the teachers who are used as health workers in schools are forced to use their class time, which is for teaching, to implement the vitamin D supplement program.” (Participant 23)

“High school students sometimes refuse to take the pill out of mischief. The goal is to improve students' physical health, especially female students, through supplementation with iron and vitamin D.” (Participant 25)

“In recent years, this program was not carried out regularly; the main reason was the incidence of COVID-19 that led to the closure of schools. Also, we did not have access to students and their parents did not cooperate to go to the health centers and receive the supplements due to the fear of COVID-19.” (Participant 2)

##### 3.1.1.1. High prevalence of VDD risk factors in Iran

Some of the main causes of VDD in Iran over the past few decades were reported as changes in socioeconomic factors, food patterns, and lifestyle (7, 19). Vitamin D deficiency varies based on age, sex, and location of residence (20). Other reasons that affect vitamin D status include the increasing prevalence of obesity, air pollution (21), urbanization rate (22), and insufficient public awareness (23).

“In the National Food and Nutrition Care Program, which is conducted by the Nutrition Research Institute in collaboration with the Nutrition Improvement Office, we witnessed that the highest prevalence of VDD was related to the provinces near the equator, because people's sun-avoidance behaviors are more common in these areas.” (Participant 12)

### 3.2. Policy stream

The policy stream focuses on how solutions can be applied for VDD prevention. The VDD prevention program was diligently implemented at the beginning of the Second National Integrated Micronutrient Survey (NIMS-II) program in 2012 (7).

“One solution is to teach people about exposure to sunlight. However, there are many cultural and social considerations against it. Now, for example, I am indoors from 7:00 am to 10:00 pm; and it is not possible for me to be exposed to sunlight. Therefore, social relations are often preferred over medical recommendations.” (Participant 8)

After that, the main solution adopted to prevent VDD in Iran was vitamin D supplementation. With the beginning of the monthly vitamin D supplementation program (50,000 IU) for female students as a pilot (2014–2015), it was established that students should receive nutrition training alongside a vitamin D supplementation plan. Following that, a monthly supplementation program with a mega-dose of vitamin D was carried out among male and female students in Iranian high schools over the course of nine academic months. In addition, since 2013, all adults, middle-aged people, and older adults have been referred to health centers to receive monthly supplements of vitamin D. However, children under 2 years of age were supplemented with A+D drops, which included 400 IU of vitamin D from the beginning of the primary health care (PHC). Health trainers in schools and healthcare workers in health centers provide nutrition education alongside vitamin D supplementation.

The next strategy was to employ a large number of nutritionists to prepare guidelines for nutrition education following Iran's Health Reform Plan in 2014. The interviewees suggested that the best solution to prevent VDD was to fortify some foods with vitamin D. Recently, a national fortification committee approved the addition of vitamin D to flour, which is currently being done in a pilot phase in some Iranian provinces.

“Supplementation is a policy that cannot be implemented for everyone. It should be done in a specific group and during a limited period. In my idea, the best policy currently being employed at the national level is the vitamin D fortification program.” (Participation 9)

“We should seek to increase vitamin D intake in the society through fortification of the dominant foods of the society.” (Participant 9)

Based on the results from the interviews and reviewing existing documents, the following activities were identified as solutions to prevent VDD in Iran: (a) the distribution of free milk in schools by prioritizing milk fortified with vitamin D, implemented in Iran between 2000 and 2010; (b) the establishment of National Food and Nutrition Care Program in 2013; and (c) the formation of the National Committee for the Prevention and Control of Non-Communicable Diseases in 2014.

The distribution of free milk in schools was implemented as a pilot in schools between 2000 and 2010. Later, vitamin D-fortified milk was supplied. However, the program was canceled in most provinces in recent years due to financial problems; it is being implemented only in eight deprived provinces since 2018. According to the results of interviews conducted in this study, a combination of strategies, including vitamin D supplementation for vulnerable groups, food fortification, and increasing public awareness could be considered as the best solutions to prevent VDD.

### 3.3. Political stream

#### 3.3.1. International and national support

The World Health Organization (WHO) plays a main role in the global governance of NCD control and prevention. The Iranian national program for the control and prevention of NCDs was drafted after the WHO published its “Global Action Plan for the Prevention and Control of NCDs 2013–2020” (24). This action plan was also discussed at the WHO regional offices (25). Any success in the prevention of NCDs requires a multi-sectoral approach, which includes the participation of all health-related sectors and relevant stakeholders from other ministries and organizations. According to the results of the interviews, it is essential to focus on prevention rather than treatment to improve Iran's “overall policies for health” developed in 2014. However, the lack of intersectoral partnership and changes in the management system caused the slow progress of the policy process.

“Regarding vitamin D fortification, it took four years to justify one of the former managers to change his point of view.” (Participant 8)

After forming a specialized working group on health and food safety in 2012 and launching the health system reform plan in 2014, more nutritionists were employed in the health sector. Further, the national program for the control and prevention of NCDs was established in 2014, and the results of the National Integrated Micronutrient Survey were publicized. These actions attracted the policymakers' attention to vitamin D policies. These streams opened the opportunity window to establish vitamin D policies by the Nutrition Improvement Office of the MoHME.

## 4. Discussion

According to the results of interviews conducted in this study, the main policy to prevent VDD in Iran was to run a supplementation strategy in children under 2 years old, high school students, and adults, as well as to refer older adults to health centers. Along with supplementation, nutrition education was identified as another program to improve vitamin D intake. However, because of the heavy workload in the educational system and the shortage of staff in healthcare, the program has not performed well. Besides, the fortification of foods with vitamin D was done as a pilot initiative in two provinces and not undertaken nationwide. Nonetheless, several food companies established vitamin D fortification voluntarily.

The agenda-setting process of this policy, obtained from the convergence of the three streams, and the presence of political entrepreneurs to join these streams led to the creation of the window of opportunity.

One of the key conclusions of this study is that treatment took precedence over prevention in the Iranian healthcare system. Hence, the major obstacles were the lack of sufficient awareness of vitamin D deficiency and the side effects of NCDs; incomplete coordination between different agencies of government; lack of statistics and evaluation; tendency toward unhealthy lifestyle, and environmental problems. In addition, the outbreak of COVID-19 and the current economic situation in many low- and middle-income countries (such as Iran) pose hurdles to the implementation of vitamin D policies despite placing them on the agenda.

Recent studies have reported several beneficial effects of higher intake of vitamin D. Vitamin D supplementation improved inflammatory and oxidative stress by reducing C-reactive protein (CRP), tumor necrosis factor-α (TNF-α), and malondialdehyde (MDA) levels (26). It also positively affected the levels of obesity indices, including body mass index (BMI), and waist circumference (WC) (27). Further, vitamin D supplementation was shown to reduce the development of depression symptoms (28).

It is important to convince politicians by providing evidence-based research to place the desired health issue on the agenda (29). In the case of vitamin D, there is a need for stakeholder engagement, as shown by national research and data on the high incidence of VDD and some evidence of its detrimental impacts on public health (7). One main challenge in this field remains the lack of national reports evaluating serum vitamin D levels after the program implementation of supplementation with mega-dose of vitamin D.

Although the results obtained in this study showed that vitamin D supplementation had a positive effect on improving vitamin D status, VDD is still considered a prevalent health problem in Iran (30). Studies conducted in other nations, such as Finland and Australia, showed that once vitamin D policies were put in place, vitamin D status increased considerably (31, 32). On the other hand, studies in some other countries showed that despite the existence of vitamin D programs, vitamin D status was undesirable (33, 34).

Numerous studies have flagged gender differences in the prevalence of VDD in Iran. One study reported that the deficiency was significantly more prevalent in girls than boys (35). Another investigation found that the highest serum 25(OH)D levels for women were lower or equivalent to the lowest values for men (36). This is in line with the results in the majority of other countries (37). However, there was no significant difference in the intake of vitamin D between girls and boys in Iran; therefore, the gender difference may be related to the lack of exposure to sunlight because of the type of clothing in Iranian girls (35). Furthermore, socioeconomic and cultural determinants have significant effects on public access to vitamin D resources (38). Besides, some factors, including obesity, physical inactivity (39), ethnicity, (40) latitude, and the amount of air pollutants, are reported to be predictors of VDD. Despite the evidence, policies do not pay enough attention to the people with special conditions, and the program is implemented equally in all parts of the country. This one-size-fits-all approach needs to be revised.

Our findings also highlight the need to focus on the role of political support at the national and international levels in setting the agenda for vitamin D policy. After developing the Global Action Plan for the Prevention and Control of NCDs 2013–2020 by the WHO, the Iranian Action Plan for the Prevention of NCDs was presented by the MoHME. The adoption of Iran's Health Reform Plan 2013 highlighted the importance of controlling NCDs (41).

After opening the window of opportunity, practical solutions and initiatives should be used. However, actors other than those in the health sector do not have enough motivation to work on this issue. Although Iran's health sector was built using a top-down strategy at the national, provincial, and urban levels (42), there is insufficient involvement from executive stakeholders and actors at lower levels in the formulation of policies. Consequently, policymakers at the top are not involved in the issues of stakeholders at the lower and local levels. Therefore, most of the policies are implemented with a lack of enthusiasm (43).

Another cause of VDD and food insecurity is the poor economic situation, which may lead to unaffordability in access to vitamin D supplements needed to implement the policy (44). Vitamin D supplementation was reported to reduce COVID-19–related deaths (45). However, based on the interviewees, the optimal implementation of the mega-dose of vitamin D supplementation program during COVID-19 faced some difficulties in terms of reduced access to students and adults. In addition to vitamin D supplementation, vitamin D fortification can be considered as a strategy to prevent VDD in Iran (46). According to the respondents, a mixed approach to reach various groups, as well as well-designed regulations with ongoing monitoring systems, can improve vitamin D status in the community.

## 5. Limitations and/strengths

To the best of the authors' knowledge, this study is the first research to identify key points to advance the implementation of vitamin D policies in Iran. Another strength was the agenda-setting approach of the study. This study provided some ideas regarding the driving forces behind the agenda-setting of vitamin D policies, which can proffer policy. However, since the study had a qualitative design and used data obtained from interviews and reviewing documents, the findings have low generalizability.

## 6. Conclusion

Applying Kingdon's agenda-setting approach, this study offered insights into the factors that influence the development of policies related to vitamin D, which could lead to the creation of preferred policy solutions. Currently, a window of opportunity for VDD prevention has been opened. Despite the emphasis of the “overall policies for health” on prevention over treatment, this is not implemented in practice due to some local problems. In addition, some challenges, such as the inadequacy of the health sector's capacity and facilities and the outbreak of COVID-19, negatively affected the implementation of the policies. Regarding political recommendations, responsible organizations such as the WHO should empower low- and middle-income countries and allocate part of the budgets for big preventative strategies. Currently, it is essential to strengthen intersectoral cooperation to better understand the problem and offer potential solutions. However, actors require political support to move this subject on the agenda. This support can be provided by the government, the Supreme Council of Health, and Food Security, the MoHME, and the Ministry of Education to retain the policy on the agenda.

## Data availability statement

The raw data supporting the conclusions of this article will be made available by the authors, without undue reservation.

## Ethics statement

The studies involving humans were approved by the Ethics Committee of Tabriz University of Medical Sciences, Tabriz, Iran (Code: IR.TBZMED.REC.1400.653). The studies were conducted in accordance with the local legislation and institutional requirements. Written informed consent for participation in this study was provided by the participants' legal guardians/next of kin.

## Author contributions

BA contributed to data collection, statistical analysis, and drafting the manuscript. BA, RK-Z, SK, and MA participated in the design and interpretation of data. All authors contributed to writing, revising, and approving the final manuscript.

## Footnotes

^1^ https://arakmu.ac.ir/file/download/regulation/1551769202-.doc

^2^https://www.gisoom.com/book/11211655/%DA%A9%D8%AA%D8%A7%D8%A8-%D9%BE%DA%98%D9%88%D9%87%D8%B4%DB%8C-%D8%AF%D8%B1-%D9%88%D8%B6%D8%B9%DB%8C%D8%AA-%D8%B1%DB%8C%D8%B2%D9%85%D8%BA%D8%B0%DB%8C-%D9%87%D8%A7%DB%8C-%D8%A7%DB%8C%D8%B1%D8%A7%D9%86-1391-%D9%BE%D9%88%D8%B1%D8%A7-2/book.
