# Correction: Vitamin D deficiency prevention policies in Iran: a retrospective policy analysis

**DOI:** 10.3389/fnut.2026.1858366

**Published:** 2026-05-01

**Authors:** Frontiers Editorial Office

**Affiliations:** Frontiers Media SA, Lausanne, Switzerland

**Keywords:** vitamin D deficiency, agenda setting, Kingdon's multiple streams, prevention, vitamin D

In the published article, there was an error in the Conflict of Interest statement. A past collaboration between the reviewer [VM] and the authors [BA, SK, and MA] was not declared.

In line with the COPE guidelines, the Handling Editor conducted a post-publication assessment that concluded that the article meets the standards for publication at Frontiers.

The correct statement appears below:

The author(s) declared that this work was conducted in the absence of any commercial or financial relationships that could be construed as a potential conflict of interest.

Reviewer VM declared a past co-authorship with authors BA, SK, and MA.

The original version of this article has been updated.

